# Genome sequences for nine *Streptococcus anginosus* strains isolated from female urogenital samples

**DOI:** 10.1128/mra.00566-25

**Published:** 2025-07-31

**Authors:** Helen Appleberry, Olive Kolar, Saad Ahmed, Omar Al Khalili, Inga Astrauskas, Adel Choubkha, Abraham Dulli, Anna Gamal, Elina Greljo, Samiah Iqbal, Neelesh Kanduri, Adam Kawamleh, Emma Kress, Walter McCain, Sophia Meza, Madison Mikolaitis, Amal Naqvi, Emily Ngo, Jackie Ostiguin, Vedant Patel, Alan J. Wolfe, Catherine Putonti, Alex Kula

**Affiliations:** 1 Department of Biology, Loyola University Chicago 224010, Chicago, Illinois, USA; 2 Bioinformatics Program, Loyola University Chicago 2456, Chicago, Illinois, USA; 3 Department of Microbiology and Immunology, Stritch School of Medicine, Loyola University Chicago 12248, Maywood, Illinois, USA; University of Maryland School of Medicine, Baltimore, Maryland, USA

**Keywords:** *Streptococcus anginosus*, urinary, urogenital

## Abstract

*Streptococcus anginosus* is a common inhabitant of the female urinary tract. Comparative genomics suggests that strains from female urogenital samples are distinct from *S. anginosus* strains isolated from other anatomical sites. Here, we present three complete and six draft genome assemblies of *S. anginosus* isolated from urine, vaginal swabs, and a perineal swab.

## ANNOUNCEMENT

The bacterium *Streptococcus anginosus* has been isolated from different human anatomical sites (see review reference 1). Recently, we found that most urinary isolates are genetically distinct (2). Because this prior analysis was based on draft genome analysis only, it prompted our study here, producing hybrid assemblies from female urogenital samples, including urine (*n* = 4), vaginal swabs (*n* = 4), and a perineal swab.

These strains were isolated from samples collected from female participants, who provided written consent, as part of prior IRB-approved studies (Table 1) (3–6). As part of these prior studies, these strains were isolated from their respective sample type via the enhanced quantitative urine culture method (3). Previously, eight of these strains were sequenced using Illumina short-read sequencing and deposited in NCBI’s SRA database (Table 1). All nine samples were requested from the Loyola Urinary Education and Research Collaborative collection and provided to the Putonti lab on Columbia nalidixic acid agar plates. Brain heart infusion (BHI) media was inoculated with single colonies from these plates and incubated at 35°C with 5% CO_2_ for 24 hours. These liquid cultures were then struck onto BHI plates and incubated under the same conditions. Plates were shipped to SeqCenter (Pittsburgh, PA, USA) for DNA extraction (ZymoBIOMICS DNA Miniprep Kit). For UMB6808, both Illumina and Nanopore sequencing were performed. Thus, from this DNA extraction, two library preparations were conducted. One library was prepared using the DNA Prep kit (Illumina) and custom IDT 10 bp unique dual indices with a target insert size of 280 bp. This library was sequenced on an Illumina NovaSeq X Plus (2 × 150 bp), and demultiplexing, quality control, and adapter trimming were performed with bcl-convert (v4.2.4). For all nine samples’ DNA extractions, Nanopore libraries were prepared using the PCR-free Oxford Nanopore Technologies Ligation Sequencing Kit (SQK-NBD114.24) with the NEBNext Companion Module (E7180L) to the manufacturer’s specifications. No additional DNA fragmentation or size selection was performed. Sequencing was performed on an Oxford Nanopore MinION Mk1B sequencer or a GridION sequencer using R10.4.1 flow cells. The run design utilized the 400 bp sequencing mode with a minimum read length of 200 bp. Adaptive sampling was not enabled. Guppy (v6.5.7) was used for super-accurate basecalling, demultiplexing, and adapter removal.

**TABLE 1:** *S. anginosus* isolate sequencing and assembly statistics^*a*^

Strain ID	Isolation source	Illumina reads (SRA accession no., No. of reads)	Nanopore reads (SRA accession no., No. of reads, N50)	Assembly accession no.	Assembly length (bp)	GC (%)	No. contigs	N50 (bp)	Coverage (short read, long read)
UMB1353	Catheterized urine^*b*^	SRR14752302 3,998,620	SRR32614275 244,043 5,113 bp	CP186002.1	1,983,438	38.77	1	1,983,438	174.369 143.562
UMB1758	Vaginal swab^*c*^	SRR26861601 1,971,830	SRR32614274 337,623 4,477 bp	GCA_033803315.2	2,066,822	38.38	2	1,624,724	131.645 141.449
UMB2004	Vaginal swab^*c*^	SRR27673562 2,277,216	SRR32614269 251,766 5,342 bp	GCA_036233835.2	2,047,298	38.97	2	1,586,917	140.295 144.852
UMB2693	Perineal swab^*c*^	SRR27673514 2,835,992	SRR32614268 185,545 5,641 bp	CP1866001.1	1,970,020	39.16	1	1,970,020	149.489 143.096
UMB3052	Voided urine^*d*^	SRR27673590 2,738,038	SRR32614267 145,817 6,461 bp	CP1866000.1	2,194,275	38.69	1	2,194,275	145.492 148.017
UMB3532	Voided urine^*c*^	SRR27673550 3,128,868	SRR32614266 311,265 4,859 bp	GCA_036235875.2	2,083,003	38.83	3	1,826,538	148.045 145.742
UMB6372	Catheterized urine^*c*^	SRR27673543 2,952,614	SRR32614265 233,170 6,013 bp	GCA_036235735.2	2,285,168	38.92	5	1,389,527	143.864 143.363
UMB6808	Vaginal swab^*d*^	SRR32614264 6,819,790	SRR32614263 265,699 5,047 bp	GCA_036236175.2	2,227,872	38.50	5 Contig_4 = plasmid^*f*^ Contig_5 = plasmid^*g*^	1,420,713	139.57 141.137
UMB8188	Vaginal swab^*e*^	SRR27673519 2,483,904	SRR32614262 323,294 5,618 bp	GCA_036234535.2	2,034,275	38.80	2	1,933,368	143.653 144.892

^*a*^ For three of the strains, complete genome sequences were produced, while the remaining six are in draft form. The sequencing of these isolates expands the publicly available collection of urogenital isolates.

^*b*^ IRB approval for study from Loyola University Chicago (206469).

^*c*^ IRB approval for study from Loyola University Chicago (207152 and 207777).

^*d*^ IRB approval for study from Loyola University Chicago (206129).

^*e*^ IRB approval for study from University of California, San Francisco (15-16363) and the University of Iowa (201608793).

^*f*^ Predicted as a plasmid based on sequence homology to accession no. CP118047.1 (query coverage = 71%; percent identity = 96.27%).

^*g*^ Predicted as a plasmid based on sequence homology to accession no. CP118037.1 (query coverage = 100%; percent identity = 100%).

Hybrid assemblies were performed using the web-tool BV-BRC (v3.36.16.5) (7) with the “auto” option. First, raw short reads were trimmed via Trim Galore (v0.6.5dev; https://github.com/FelixKrueger/TrimGalore) and normalized using BBNorm (v 19 October 2017; https://jgi.doe.gov/data-and-tools/software-tools/bbtools/). Long reads were quality controlled and filtered using filtlong (v0.2.1; https://github.com/rrwick/Filtlong). The filtered reads were assembled using Unicycler v0.4.8 (8), and polishing was performed by two rounds of racon v1.4.20 (9) followed by two rounds of pilon v1.23 (10). As part of the assembly process, the circularized chromosome contigs were automatically rotated to begin with *dnaA*. Genome assemblies were annotated using the BV-BRC tool to confirm species designation only and then annotated upon its deposit to NCBI via PGAP (v6.9) (11). Genome assemblies were queried against the NCBI core_nt nucleotide database to identify putative plasmid sequences. Unless otherwise specified, default parameters were used for all software tools.

## Data Availability

Read and assembly data are publicly available through NCBI, and the accession numbers are listed in Table 1.
